# Structure of a Nudix hydrolase (MutT) in the Mg^2+^-­bound state from *Bartonella henselae*, the bacterium responsible for cat scratch fever

**DOI:** 10.1107/S1744309111011559

**Published:** 2011-08-16

**Authors:** Garry W. Buchko, Thomas E. Edwards, Jan Abendroth, Tracy L. Arakaki, Laura Law, Alberto J. Napuli, Stephen N. Hewitt, Wesley C. Van Voorhis, Lance J. Stewart, Bart L. Staker, Peter J. Myler

**Affiliations:** a Seattle Structural Genomics Center for Infectious Disease, http://www.ssgcid.org, USA; b Biological Sciences Division, Pacific Northwest National Laboratory, Richland, Washington, USA; c Emerald BioStructures, Bainbridge Island, Washington, USA; d Department of Medicine, University of Washington, Seattle, Washington, USA; e Seattle Biomedical Research Institute, Seattle, Washington, USA; f Department of Medical Education and Biomedical Informatics and Department of Global Health, University of Washington, Seattle, Washington, USA

**Keywords:** Nudix hydrolases, *Bartonella henselae*, MutT, cat scratch fever

## Abstract

Cat scratch fever (also known as cat scratch disease and bartonellosis) is an infectious disease caused by the proteobacterium *Bartonella henselae* following a cat scratch. Although the infection usually resolves spontaneously without treatment in healthy adults, bartonellosis may lead to severe complications in young children and immunocompromised patients, and there is new evidence suggesting that *B. henselae* may be associated with a broader range of clinical symptoms then previously believed. The genome of *B. henselae* contains genes for two putative Nudix hydrolases, BH02020 and BH01640 (KEGG). Nudix proteins play an important role in regulating the intracellular concentration of nucleotide cofactors and signaling molecules. The amino-acid sequence of BH02020 is similar to that of the prototypical member of the Nudix superfamily, *Escherichia coli* MutT, a protein that is best known for its ability to neutralize the promutagenic compound 7,8-dihydro-8-oxoguanosine triphos­phate. Here, the crystal structure of BH02020 (*Bh*-MutT) in the Mg^2+^-bound state was determined at 2.1 Å resolution (PDB entry 3hhj). As observed in all Nudix hydrolase structures, the α-helix of the highly conserved ‘Nudix box’ in *Bh*-­MutT is one of two helices that sandwich a four-stranded mixed β-sheet with the central two β-strands parallel to each other. The catalytically essential divalent cation observed in the *Bh*-MutT structure, Mg^2+^, is coordinated to the side chains of Glu57 and Glu61. The structure is not especially robust; a temperature melt obtained using circular dichroism spectroscopy shows that *Bh*-­MutT irreversibly unfolds and precipitates out of solution upon heating, with a *T*_m_ of 333 K.

## Abstract 2

*B. henselae* is the etiological agent responsible for cat scratch fever (bartonellosis). The crystal structure of the smaller of the two Nudix hydrolases encoded in the genome of *B. henselae*, *Bh*-MutT, was determined to 2.1 Å resolution.

## 1. Introduction

Cat scratch fever (also known as cat scratch disease and bartonell­osis) is a bacterial infection characterized by a bump or blister at the original site of entry, swollen lymph nodes, headaches and overall general discomfort. The etiological agents responsible for the disease are bacteria from the fastidious hemotropic Gram-negative genus *Bartonella* (Chomel & Kasten, 2010 ▶). This genus contains 19 species, of which six have been associated with the human disease (Florin *et al.*, 2008 ▶). Of these six species, *B. henselae* is the primary agent responsible for cat scratch fever. As the name of the disease suggests, the chief reservoir for *B. henselae* is the domestic cat, with the bacteria being transmitted to humans primarily *via* scratches (Florin *et al.*, 2008 ▶). It is debatable whether cats themselves become ill owing to *B. henselae* infection (Chomel *et al.*, 1996 ▶). There is no horizontal transfer of *B. henselae* between cats; instead, inter-feline spread of *B. henselae* occurs through a vector, *Ctenocephalides felix*, the cat flea (Chomel *et al.*, 1996 ▶). It was generally believed that the infection did not require medical treatment, except in immunocompromised individuals, including those with human immunodeficiency virus infections. However, as techniques to diagnosis the presence of *B. henselae* have improved, there is a new concern that it may be responsible for a broader range of clinical symptoms, including prolonged fever of unknown origin (Florin *et al.*, 2008 ▶). Owing to the widening spectrum of *B. henselae* infections and the widespread presence of *B. henselae* in the domestic cat, there is a renewed interest in understanding the biochemistry of *B. henselae* and in developing new therapies to combat this organism.

**Figure 1:**
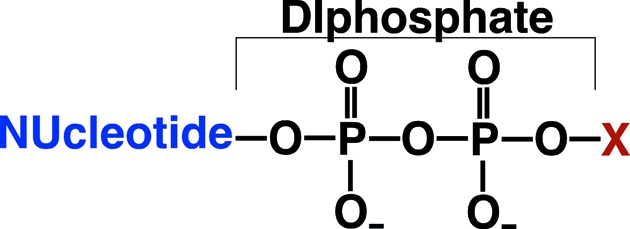
General chemical structure of a Nudix substrate: a **Nu**cleotide **di**phosphate linked to a moiety ‘**x**’. The moiety ‘*x*’ may be as small as a hydrogen atom or metal ion.

The genome of *B. henselae* contains genes for two putative Nudix hydrolases, BH02020 and BH01640 (KEGG). The Nudix superfamily of proteins (Pfam PF00293) is large, with over 16 000 members as of the end of 2010, and widely distributed, with members identified in organisms as diverse as viruses and humans (McLennan, 2006 ▶). Associated with their wide distribution is an ability to act, primarily as polyphosphate pyrophosphohydrolases (Bessman *et al.*, 1996 ▶), on a wide range of substrates of the general structure shown in Fig. 1 ▶. It is from this general structure, **Nu**cleoside **di**phosphates linked to some other moiety **x**, that the acronym ‘Nudix’ arose (Abeygunawardana *et al.*, 1995 ▶; Bessman *et al.*, 1996 ▶). Identified Nudix substrates include capped mRNA (Wang, Jiao *et al.*, 2002 ▶), dinucleotide coenzymes, nucleotide sugars, nucleotide alcohols, dinucleotide polyphosphates, both canonical and oxidized (deoxy)­ribonucleoside triphosphates (NTPs) (Dunn *et al.*, 1999 ▶) and canonical (deoxy)ribonucleoside diphosphates (NDPs) (Fisher *et al.*, 2004 ▶; Buchko *et al.*, 2008 ▶). Some Nudix substrates have been reported without a nucleoside, such as thiamine pyrophosphate (Lawhorn *et al.*, 2004 ▶), diphosphoinositol polyphosphates (Safrany *et al.*, 1998 ▶) and dihydroneopterin triphos­phate (Klaus *et al.*, 2005 ▶). With a few exceptions, catalysis depends on a highly conserved sequence of amino acids, G*X*_5_E*X*_7_RE*U* *X*EE*X*G*U* (where *U* is an aliphatic and hydrophobic amino acid and *X* is any amino acid), called the ‘Nudix box’ (Abeygunawardana *et al.*, 1995 ▶; Bessman *et al.*, 1996 ▶). This box forms a loop–helix–loop structural motif that is part of the larger substrate-binding Nudix fold. Glutamic acid residues in the core of the Nudix box, RE*UX*EE, bind one to three divalent cations, and at least one cation is required for hydrolase activity. While Nudix hydrolases can bind a number of different divalent cations, including Mn^2+^ and Co^2+^ (Lin *et al.*, 1997 ▶; Buchko *et al.*, 2008 ▶), the highest activity observed *in vitro* is usually with Mg^2+^, the cation that is most likely to be relevant under physiological conditions (Bessman *et al.*, 1996 ▶). Detailed features of the Nudix fold, a mixed β-sheet sandwiched between the catalytic α-helix of the Nudix box and a second α-helix, confer the substrate specificity of Nudix hydrolases (Mildvan *et al.*, 2005 ▶; McLennan, 2006 ▶). By hydrolyzing their substrates, Nudix proteins effect the elimination of potentially toxic endogenous nucleotides from the cell and regulate the concentration of nucleotide cofactors and signaling molecules inside the cell (Bessman *et al.*, 1996 ▶; Galperin *et al.*, 2006 ▶). For example, *Escherichia coli* MutT protein, the prototypical member of the Nudix superfamily, preferably hydrolyzes 7,8-dihydro-8-oxo­guanosine triphosphate, a promutagenic compound that is generated during normal cellular metabolism and upon exposure to oxidative stress (Grollman & Moriya, 1993 ▶), into nonmutagenic nucleoside monophosphate and inorganic phosphate (Maki & Sekiguchi, 1992 ▶; Weber *et al.*, 1992 ▶). Given the important housekeeping duties performed by Nudix hydrolases, the crystal structure of one of the two Nudix hydrolases present in the genome of *B. henselae*, BH02020 (*Bh*-­MutT), was solved and its structure described in relation to other Nudix protein structures.

## 2. Materials and methods

### 2.1. Cloning, expression and purification

The *Bh*-MutT gene (BH02020/YP_033055.1; NCBI) was amplified using the genomic DNA of *B. henselae* strain Houston-1 and the oligonucleotide primers 5′-GGGTCCTGGTTCGATGCCTATAAA­AAGTTCACTTCTTATT-3′ (forward) and 5′-CTTGTTCGTG­CTGTTTATTAAATAAAGAAATTTTTTAATACCTGAACTA-3′ (reverse) (Invitrogen, Carlsbad, California, USA). The amplified Bh-*­MutT* gene was then inserted into *Nru*I/*Pme*I-digested expression vector AVA0421 by ligation-independent cloning at a site that provided a 21-residue tag (MAHHHHHHMGTLEAQTQGPGS–) at the N-terminus of the expressed protein (Choi *et al.*, 2011 ▶). The recombinant plasmid was transformed into *E. coli* BL21(DE3)-R3-pRARE2 cells (a gift from SGC-Toronto, Toronto, Ontario, Canada) *via* a heat-shock method and the resulting clone was used to inoculate 2 l of autoinduction medium (Studier, 2005 ▶) in a LEX bioreactor (Harbinger Biotechnology and Engineering Corporation, Markam, Ontario, Canada). After 72 h of growth at 293 K, the cells were harvested, flash-frozen in liquid nitrogen and stored at 193 K. The cell pellet was subsequently solubilized in 200 ml lysis buffer (25 m*M* HEPES, 500 m*M* NaCl, 5% glycerol, 30 m*M* imidazole, 0.5% CHAPS, 10 m*M* MgCl_2_, 1 m*M* TCEP, 1 m*M* AEBSF, pH 7.0) containing 10 mg lysozyme and sonicated for 30 min. After sonication, samples were treated with Benzonase (500 units) and centrifuged for 1 h (14 000 rev min^−1^ in a Sorvall SLA-1500 rotor) to remove cell debris. The protein was purified by immobilized metal-ion affinity chromatography on a 5 ml HisTrap FF Nickel Sepharose column (GE Healthcare, Piscataway, New Jersey, USA) using an ÄTKAexplorer (GE Healthcare, Piscataway, New Jersey, USA). Fractions containing *Bh*-MutT were pooled, concentrated and further purified by size-exclusion chromatography on a Superdex75 HiLoad 26/60 column (GE Healthcare, Piscataway, New Jersey) that also exchanged the protein into crystallization buffer (25 m*M* HEPES, 500 m*M* NaCl, 5% glycerol, 2 m*M* dithiothreitol, 0.025% sodium azide pH 7.5). Fractions containing *Bh*-MutT were pooled and con­centrated (Amicon Ultra-15 with a 3000 Da MWCO) to 27 mg ml^−1^. Protein samples were aliquoted in 100 µl volumes into PCR tubes, flash-frozen in liquid nitrogen and stored at 193 K (Deng *et al.*, 2004 ▶). For the circular dichroism and nuclear magnetic resonance spectroscopy experiments, ^15^N-labeled uncleaved *Bh*-MutT was prepared from 1 l of autoinduction medium (Studier, 2005 ▶) and purified by a conventional two-step protocol involving Ni- NTA affinity chromatography followed by size-exclusion chromatography using CD buffer (100 m*M* NaCl, 20 m*M* Tris, 1.0 m*M* dithiothreitol, pH 7.1).

### 2.2. Nuclear magnetic resonance spectroscopy

A Varian 600-Inova spectrometer equipped with a ^1^H{^13^C,^15^N} triple-resonance probe and pulse-field gradients was used to collect a 2D ^1^H–^15^N HSQC spectrum from ^15^N-labeled *Bh*-MutT in CD buffer (293 K).

### 2.3. Circular dichroism spectroscopy

A calibrated Aviv Model 410 spectropolarimeter (Lakewood, New Jersey, USA) was used to collect circular dichroism data from a 0.05 m*M* sample of *Bh*-MutT in CD buffer in a quartz cell of 0.1 cm path length. Using the same sample, a thermal denaturation curve was obtained by recording the ellipticity at 220 nm in 2.0 K intervals from 283 to 353 K. A quantitative estimation of the melting temperature, *T*_m_, was obtained by taking the first derivative of the thermal denaturation curve (Greenfield, 2006 ▶) using the *Aviv* software. Steady-state wavelength spectra for *Bh*-MutT were recorded in 0.5 nm increments between 200 and 260 nm at 298 K. The reported steady-state wavelength spectrum was the result of averaging two consecutive scans with a bandwidth of 1.0 nm and a time constant of 1.0 s. The spectrum was processed by subtracting a blank spectrum from the protein spectrum and then automatically line-smoothing the data using the *Aviv* software.

### 2.4. Crystallization

Sitting-drop vapor-diffusion crystallization trials were set up at 289 K using the Wizard full matrix (Emerald BioSystems, Bainbridge, Washington, USA) or ProPlex sparse-matrix (Molecular Dimensions, Newmarket, England) screens with or without *in situ* proteolysis with chymotrypsin (0.01 mg ml^−1^; Wernimont & Edwards, 2009 ▶). *Bh*-­MutT stock solutions (0.4 µl) were mixed with reservoir solution (0.4 µl) and equilibrated against reservoir solution (100 µl) using 96-­well Compact Jr plates (Emerald BioSystems, Bainbridge, Washington, USA). Crystals grew in several conditions, but those used in X-ray data collection and structure determination were obtained from ProPlex condition B5 without *in situ* proteolysis (27 mg ml^−1^ protein against 0.1 *M* HEPES, pH 7.5, 10% PEG 4000, 0.1 *M* MgCl_2_).

### 2.5. Data collection and structure refinement

A crystal of *Bh*-MutT was cryoprotected with reservoir solution supplemented with 20% glycerol, flash-frozen in liquid nitrogen, stored under liquid nitrogen, and shipped to the Advanced Light Source (ALS) at Lawrence Berkeley National Laboratory for X-ray data collection. A data set was collected on ALS beamline 5.0.3 with an ADSC Quantum 315 detector (Table 1 ▶) and reduced with *XDS*/*XSCALE* (Kabsch, 2010 ▶). The structure was solved by molecular replacement using *Phaser* (McCoy *et al.*, 2007 ▶) from the *CCP*4 suite (Winn *et al.*, 2011 ▶) with the NMR solution structure of *E. coli* MutT (PDB entry 1tum, first model; Lin *et al.*, 1997 ▶) as the search model after alignment modification in *CHAINSAW* (Stein, 2008 ▶). The structure was initially rebuilt with *Buccaneer* (Cowtan, 2006 ▶) followed by additional model building in *ARP*/*wARP* (Langer *et al.*, 2008 ▶). The final model was produced after numerous iterative rounds of refinement in *REFMAC* (Murshudov *et al.*, 1997 ▶) and manual building in *Coot* (Emsley & Cowtan, 2004 ▶). The final model showed good geometry and fitness (Table 1 ▶) according to analysis with *MolProbity* (Chen *et al.*, 2010 ▶).

The amino-acid sequence of *Bh*-MutT deposited in the PDB is numbered sequentially, Met1–Ile158, starting with the 21 non-native residues that were part of the N-terminal polyhistidine tag. Here, the first native residue, Met22 in the PDB deposition, is labeled Met1.

## 3. Results and discussion

### 3.1. Structure of *Bh*-MutT

**Figure 2:**
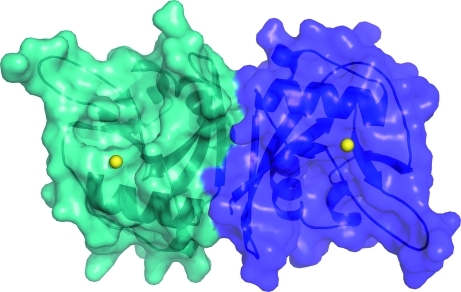
A transparent surface representation of the two subunits in the asymmetric unit of the crystal structure of *Bh*-MutT (PDB entry 3hhj). A cartoon representation of the structure is illustrated underneath the surface. The two subunits are colored cyan and purple and the Mg^2+^ ions are colored yellow.

The crystal structure includes two molecules of *Bh*-MutT, *A* and *B*, packed together in the asymmetric unit. Electron density was missing at the N-terminus for the 21-residue tag plus the first six native residues in both subunits. At the C-terminus, electron density was missing for the last two residues of subunit *A* and the last residue of subunit *B*. The structure of each molecule in the asymmetric unit was similar; the C^α^ atoms of residues Ser6–Phe135 of the two subunits superposed with a root-mean-square deviation (r.m.s.d.) of 0.61 Å (Winn *et al.*, 2011 ▶). With a buried surface area of 11.4%, the PDBe *PISA* server (http://www.ebi.ac.uk/msd-srv/prot_int/pistart.html) predicts the biological unit of the protein to be a dimer as shown in Fig. 2 ▶. This observation in the crystalline state was also corroborated in solution. *Bh*-MutT eluted from a Superdex75 size-exclusion column with a retention time of 68 min, a value that is more consistent with a 36 kDa dimer than an 18 kDa monomer (21-residue N-­terminal tag present). Furthermore, the ^1^H–^15^N HSQC spectrum of uncleaved ^15^N-labeled *Bh*-MutT featured broad cross-peaks characteristic of a protein much larger than 18 kDa (data not shown). Dimeric Nudix hydrolases have previously been observed both in solution and in the crystalline state and are sometimes required for the Nudix hydrolase to function optimally (Wang, Mura *et al.*, 2002 ▶).

**Figure 3:**
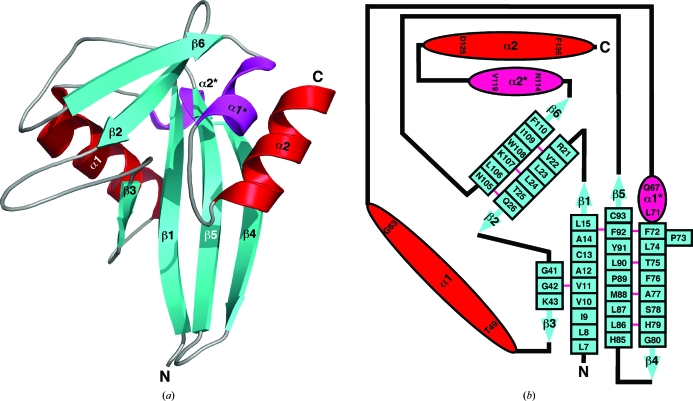
(*a*) Cartoon representation of the crystal structure of *Bh*-MutT in the Mg^2+^-bound state (PDB entry 3hhj). The β-strands are colored blue, α-helices red and 3_10_-helices magenta. The 23-residue ‘Nudix box’ consists of residues Gly42–Val64. (*b*) Secondary-structure diagram of *Bh*-MutT. The α-helices are drawn as red ovals and 3_10_-helices as magenta ovals, with the residue number of the beginning and the end of each element shown. The β-strands are drawn as solid blue arrows with each residue indicated within a box. The β-sheets contain a bulge at residue Pro73. A solid pink line between β-strand residues indicates dual hydrogen bonds between two residues in an antiparallel β-­sheet. The regions between secondary-structure elements (black lines) are not drawn to scale.

Fig. 3 ▶(*a*) shows a cartoon representation of the structure of *Bh*-­MutT and Fig. 3 ▶(*b*) shows a two-dimensional schematic of the individual secondary structure components. The molecule is com­posed of six β-strands organized into two β-sheets, two α-helices and two 3_10_-helices. Four of the β-strands form the core of the Nudix fold, a mixed β-sheet with the central two β-strands parallel to each other, sandwiched between the catalytic helix (α1) and an orthogonally orientated C-terminal helix (α2). Such an arrangement is observed in Nudix proteins of known structure (Mildvan *et al.*, 2005 ▶; McLennan, 2006 ▶).

**Figure 4:**
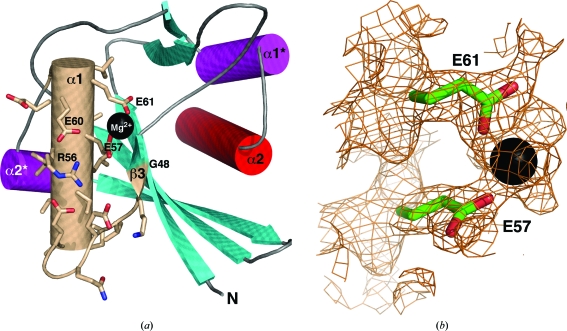
(*a*) Cartoon representation of the crystal structure of *Bh*-MutT with the 23-residue Nudix box highlighted in wheat. The side chains of the residues that form the Nudix box are shown (O, red; N, blue) along with the Mg^2+^ ion (black sphere). (*b*) The electron density surrounding the Mg^2+^ ion in the crystal structure of *Bh*-MutT illustrated at 1.2σ. The O atoms from the side chains of Glu61 and Glu57 are coordinated to the ion.

Nudix proteins are all characterized by the presence of a highly conserved 23-residue ‘Nudix box’, G*X*_5_E*X*_7_RE*UX*EE*X*G*U*, where *U* is a bulky hydrophobic group and *X* is any residue (Abeygunawar­dana *et al.*, 1995 ▶; Bessman *et al.*, 1996 ▶). The Nudix sequence adopts a loop–helix–loop motif (Koonin, 1993 ▶) that serves to co­ordinate the catalytically essential divalent cation (usually Mg^2+^) to the protein (Lin *et al.*, 1997 ▶; Bailey *et al.*, 2002 ▶; Gabelli *et al.*, 2001 ▶). Nine of the 23 residues in the Nudix box are highly conserved, **G** ^42^ *X*_5_ **E** ^48^ *X*_7_ **R** ^56^ **E** ^57^ **L** ^58^ *X* **E** ^60^ **E** ^61^ *X* **G** ^63^ **V** ^64^ (bold, with the superscripts referring to the residue numbers in the *Bh*-MutT sequence), and Fig. 4 ▶(*a*) illustrates the relative orientation of these highly conserved residues in the loop–helix–loop Nudix motif of *Bh*-MutT. As observed in other Nudix structures, all of the polar conserved residues in the α-helix of *Bh*-MutT are orientated on the same side and are opposite the central mixed β-sheet. A small hydrophobic pocket formed by the side chains of Leu58 and Val64 is likely to play a conserved role in stabilizing the loop–helix–loop Nudix motif. The similar three-dimensional orientations of the conserved residues in the Nudix box of all known Nudix protein structures suggest that these residues play similar roles in all Nudix hydrolases, binding one or more divalent cations (Wang, Mura *et al.*, 2002 ▶). This is true for *Bh*-MutT, as shown in the electron-density map in Fig. 4(*b*), in which the hydroxyl groups of Glu57 and Glu61 are near the Mg^2+^ ion at distances of 2.10 and 2.11 Å, respectively, which are within the expected Mg^2+^-coordination distance range (Zheng *et al.*, 2008 ▶). It has been established that binding of the catalytic divalent metal ion occurs through the side chains of the glutamic acid residues in the loop–helix–loop Nudix motif (Lin *et al.*, 1997 ▶; Bailey *et al.*, 2002 ▶; Gabelli *et al.*, 2001 ▶; Buchko *et al.*, 2008 ▶). The bound metal in turn complexes with the diphosphate part of the substrate (Fig. 1 ▶) and forms part of the enzyme’s active site (Lin *et al.*, 1997 ▶). Diphosphate interactions with both the loop–helix–loop Nudix motif and the divalent metal cation are features that are common to all Nudix proteins. The other parts of the Nudix substrate, the nucleoside and the moiety ‘*x*’, interact with the side chains and motifs elsewhere in the protein (Lin *et al.*, 1997 ▶; Gabelli *et al.*, 2001 ▶; Bailey *et al.*, 2002 ▶; Massiah *et al.*, 2003 ▶; Mildvan *et al.*, 2005 ▶). Differences in the shape and nature of the environment surrounding the Nudix box are responsible for the different substrate specificities of different Nudix hydrolases and some of the structural differences between Nudix hydrolases (Ranatunga *et al.*, 2004 ▶).

### 3.2. Circular dichroism profile and thermal stability of *Bh*-MutT

**Figure 5:**
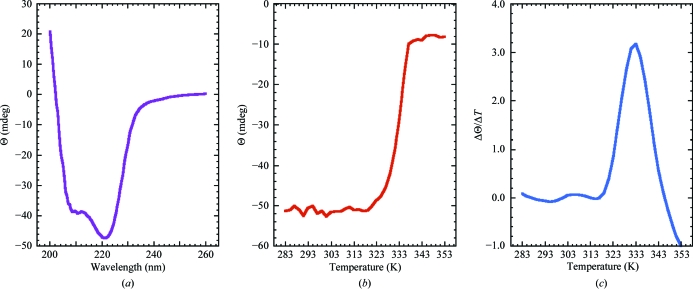
(*a*) Circular dichroism steady-state wavelength spectrum for *Bh*-MutT (0.05 m*M*) in CD buffer collected at 298 K. (*b*) The CD thermal melt for *Bh*-MutT obtained by measuring the ellipticity at 220 nm in 2.0 K intervals between 283 and 353 K. (*c*) The first derivative of the thermal melt curve shows that the protein has a melting temperature of 333 K.

Circular dichroism spectroscopy is sensitive to small changes in a protein’s backbone and consequently it is a useful tool to quickly probe the conformation of proteins in solution and assess the effect of variables on a protein’s structure (Woody, 1974 ▶). Fig. 5 ▶(*a*) shows the steady-state CD spectrum for *Bh*-MutT collected at 298 K. The dominant feature of the spectrum is characteristic of an α-helical secondary structure: a double minimum at approximately 222 and 208 nm and an extrapolated maximum around 195 nm (Holzwarth & Doty, 1965 ▶; Greenfield, 2006 ▶). Note that the double minimum is skewed and more intense around 220 nm, which is likely to be a consequence of the contribution of other secondary-structure elements to the CD steady-state spectrum.

Usually, the ellipticity at a specific wavelength increases with increasing temperature as the protein unfolds and a melting tem­perature, *T*_m_, may be estimated for the transition between a structured and an unstructured state (Karantzeni *et al.*, 2003 ▶). However, it has recently been observed that the thermal melt for the Nudix hydrolase DR_0079 from the bacterium *Deinococcus radiodurans* displays the opposite behavior, suggesting an increase in ordered structure with an increase in temperature (Buchko, 2010 ▶). To probe whether this unusual temperature effect observed for DR_0079 is more widespread in Nudix hydrolases, a melting temperature was determined for *Bh*-MutT. As shown in Fig. 5 ▶(*b*), the melting profile was typical. A gradual increase in ellipticity at 220 nm was observed for *Bh*-MutT up to ∼323 K, followed by a more rapid increase in ellipticity that leveled off at ∼335 K. Visual inspection of the sample after heating to 353 K showed evidence of precipitation, indicating that the unfolding was irreversible, and consequently the CD data could not be analyzed thermodynamically (Karantzeni *et al.*, 2003 ▶). A quantitative estimation of the *T*_m_ for this transition was still possible by assuming a two-state model and taking a first derivative of the curve in Fig. 5 ▶(*b*) (Greenfield, 2006 ▶). The maximum of this first derivative, shown in Fig. 5 ▶(*c*), is 333 K.

## 4. Conclusions

The crystal structure of *Bh*-MutT is similar to the structures reported for other Nudix hydrolases. The α-helix of the highly conserved Nudix box in *Bh*-MutT is one of two helices that sandwich a four-stranded mixed β-sheet with the central two β-strands parallel to each other. The structure was determined in the metal-bound form, with the catalytically essential divalent cation Mg^2+^ observed to be co­ordinated to the side chains of Glu57 and Glu61. While the crystal structure of *Bh*-MutT is similar to those of other Nudix hydrolases, these enzymes act on a wide range of substrates. The structural diversity that surrounds the basic Nudix box structure confers the functional diversity of Nudix hydrolases (Ranatunga *et al.*, 2004 ▶; Mildvan *et al.*, 2005 ▶; McLennan, 2006 ▶). The next step in understanding the cellular bio­chemical role of *Bh*-MutT in *B. henselae* is to equate the enzymatic activity of *Bh*-MutT to specific Nudix substrates (Ranatunga *et al.*, 2004 ▶; Buchko *et al.*, 2008 ▶). Such information, together with the structure provided here for *Bh*-MutT, will make it possible to better correlate Nudix sequences and structures with specific substrates and facilitate rational structure-based drug design targeting *B. henselae.*

## Supplementary Material

PDB reference: Nudix hydrolase, 3hhj

## Figures and Tables

**Table 1:** Summary of the diffraction data-collection and refinement statistics for *Bh*-MutT Value in parentheses are for the highest resolution shell.

Data collection	
X-ray source	ALS beamline 5.0.3
Detector	ADSC Q315 CCD
X-ray wavelength (Å)	0.97946
Temperature (K)	100
Data set	Native
Space group	*C*2
Unit-cell parameters	
*a* (Å)	96.50
*b* (Å)	93.49
*c* (Å)	43.88
α = γ (°)	90
β (°)	109.24
Matthews coefficient (Å^3^ Da^−1^)	2.62
Solvent content (%)	53.12
Resolution range (Å)	50–2.1 (2.15–2.10)
Mean *I*/σ(*I*)	12.71 (2.06)
No. of observed reflections†	21194 (1406)
Completeness (%)	98.4 (98.2)
Multiplicity	3.6 (2.6)
*R*_merge_‡ (%)	0.073 (0.488)
*R*_meas_§ (%)	0.086 (0.606)
Phasing	
Molecular replacement model	1tum
Refinement	
No. of reflections used	20108
*R*_work_¶ (%)	20.2 (27.7)
*R*_free_†† (%)	23.4 (31.5)
Mean *B* factor (Å^2^)	30.5
R.m.s.d. bonds (Å)	0.014
R.m.s.d. angles (°)	1.391
Model validation	
*MolProbity* Ramachandran analysis	
Most favored (%)	98
Additionally allowed (%)	2
*MolProbity*	
Clash score, all atoms [percentile]	7.59 [94th]
*MolProbity* score [percentile]	1.82 [90th]

† All reflections were used in data reduction.

‡ *R*_merge_ = 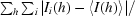 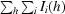.

§ *R*_meas_ = 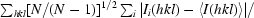 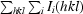 (Diederichs & Karplus, 1997 ▶).

¶ *R*_work_ = 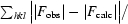 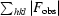, where *F*_obs_ and *F*_calc_ are the observed and calculated structure factors, respectively, calculated with the 95% of the reflections remaining after setting aside 5% for *R*_free_.

†† *R*_free_ = 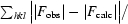 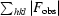. The free *R* factor was calculated using 5% (1086) of the reflections, which were omitted from the refinement.
